# Prescribing Trend of Antibiotics Among the Patients Admitted in Intensive Medical Care Unit: A Prospective Observational Study

**DOI:** 10.7759/cureus.67101

**Published:** 2024-08-18

**Authors:** Jegatheeswaran M, Mohammad Nezamuddin Khan, Ajith R, Sarumathy Sundararajan, Nanda Kumar R

**Affiliations:** 1 Department of Pharmacy Practice, SRM (Sri Ramaswamy Memorial) College of Pharmacy, Faculty of Medical and Health Sciences, SRM Institute of Science and Technology, Chengalpattu, IND; 2 Department of General Medicine, SRM (Sri Ramaswamy Memorial) Medical College Hospital and Research Centre, Chengalpattu, IND

**Keywords:** imcu, antibiotics, prescribing trend, occupancy index, defined daily dose, intensive medical care unit

## Abstract

Background: Intensive medical care units (IMCUs) usually admit patients who are in critical medical need and require the utmost attention of healthcare professionals, along with the best treatment options available. These patients are prone to infections and require various antibiotics for the treatment. Varying costs of antibiotics, variable dosage forms, and antibiotic resistance cause an economic burden on patients

Methodology: This study was designed and conducted prospectively to evaluate the prescribing pattern of antibiotics at the IMCU in a tertiary care hospital. A total of 102 patients were included in the study based on the exclusion and inclusion criteria, and the collected data was tabulated in an Excel sheet and analyzed using Prism GraphPad software. Data were presented as numbers and percentages.

Results: Most of the patients were in the age group of 41-50 years. The number of male patients was slightly more than that of female patients. The majority of the patients admitted to the IMCU had acute pulmonary edema and cerebrovascular accidents. Most antibiotics were prescribed empirically and administered parenterally, of which Amoxicillin + Potassium clavulanate was the most commonly used antibiotic. Tigecycline had the highest daily defined dose per 100 bed days value, and injection Sulbactam + Cefoperazone was the costliest of all antibiotic therapy.

Conclusion: Antibiotic therapy used in the IMCU consisting of Sulbactam + Cefoperazone was found to be costlier, and Amoxicillin + Potassium clavulanate were the commonly prescribed antibiotics among the other prescribed antibiotics. The average cost of antibiotics was found to be higher, which increased the economic healthcare burden for patients and their families.

## Introduction

Emergency care at the intensive medical care unit (IMCU) is a critical aspect of modern healthcare. Patients in intensive care units (ICUs) require specialized care, including advanced life support, continuous monitoring, and specialized medical treatment [1]. The emergency care of these patients requires a coordinated and multidisciplinary approach, with healthcare professionals working together to provide timely and effective care. ICU patients are often severely sick and may undergo unexpected changes in their state, including breathing difficulties, cardiac arrest, or infection. Recognizing these changes quickly is critical for delivering prompt and effective therapy, which may improve patient outcomes [2].

The treatment of diseases in the IMCU aims to stabilize and improve the patient's condition and prevent further deterioration. Common treatments include administering medications such as antibiotics, painkillers, diuretics, and anticoagulants, depending on the disease, via oral, intravenous, or other routes [3]. Oxygen therapy and mechanical ventilation are provided for respiratory issues, while fluid management is essential for maintaining fluid balance [4]. Continuous monitoring by healthcare professionals ensures appropriate adjustments to treatments, and nutrition support, either enteral or parenteral, helps maintain health. Specific antibiotics used at the IMCU against infections are penicillin or amoxicillin for streptococcal infections, azithromycin or doxycycline for respiratory infections, topical mupirocin or oral clindamycin for skin infections, and ciprofloxacin or metronidazole for gastrointestinal infections [5].

Patients admitted to IMCUs are prone to deterioration of health and death due to frequent initial and subsequent infections during their stay. The Extended Prevalence of Infection in Intensive Care study elucidated that 51% of patients with gram-negative bacteria (GNB) isolation were infected, accounting for 62% of infectious episodes [6]. Resistance to antimicrobials is a growing concern in ICUs globally, with gram-positive infections being common in developed countries. Nosocomial bacteremia and hospital-acquired pneumonia studies have shown that resistance to therapeutic antibiotics correlates with poor patient outcomes, although the full clinical impact is still uncertain [7].

The WHO has issued guidelines for antibiotic use in healthcare settings, emphasizing proper diagnosis, appropriate selection, and optimal dosing and duration, along with the importance of stewardship programs to ensure rational use, as irrational use of antibiotics can lead to antimicrobial resistance [8]. The selection of appropriate antibiotics in IMCUs is crucial, as improper use can lead to antibiotic-resistant infections. To optimize antibiotic usage and minimize resistance, antibiotic stewardship programs are recommended. A systematic review demonstrated that these stewardship programs in ICUs reduce therapy duration, decrease *Clostridioides difficile* infections, and improve patient outcomes [9].

Antimicrobial resistance in ICUs is a serious health issue affecting patient outcomes globally. Bacterial infections like sepsis and encephalitis are becoming harder to treat as bacteria develop resistance, reducing treatment efficacy. This resistance leads to longer hospital stays, increased healthcare costs, worse treatment outcomes, and higher mortality rates. In Europe, antibiotic resistance costs approximately €9 billion annually, with 25,000 deaths attributed to multidrug-resistant bacterial infections each year [10].

In the IMCU, antibiotic selection depends on the patient's clinical presentation, suspected infection source, and local resistance patterns. Empiric therapy should be initiated promptly, guided by regional susceptibility trends and likely pathogens, with input from a multidisciplinary team including ICU staff, infectious disease specialists, and microbiologists [8].

This study was conducted to evaluate antimicrobial medication use in the IMCU, focusing on antibiotic prescription habits such as selection, dosage, frequency, duration, and adherence to standards. It was also done to determine the daily defined doses of the antibiotics used as well as the cost of the antibiotic therapy.

## Materials and methods

A six-month prospective observational study was designed and conducted to assess the utilization of antibiotics, sensitivity, and cost in the Intensive Medical Care Unit (IMCU) of SRM Medical College Hospital and Research Centre, Chennai, Tamil Nadu. The study included a total of 102 participants (the sample size was determined using Raosoft (Raosoft Inc, Seattle, Washington) software based on the 95% confidence interval, 4.99% margin of error, and 15% contingency and also taking into account the inpatient occupancy rate) of age ≥ 18 years who were prescribed at least one antibiotic and who gave consent to be a part of the study. Patients who were already on antibiotics, those transferred or discharged before completion of the study, and those unwilling to participate were excluded. Information was collected from patient records and follow-up records. Demographic details, clinical characteristics, antibiotic use (including indication, dosage, frequency, route of administration, and cost), and antibiogram sensitivity patterns were recorded in a specially designed data collection form. Antibiotic usage was analyzed using the defined daily dose (DDD) and occupancy index methods, and costs were calculated based on hospital pharmacy data.

Determination of occupancy index

Occupancy index is determined in order to analyze the growth pattern of inpatient admissions in a hospital setting. It is calculated to manage the occupancy rates and to avoid overcrowding.

Occupancy index: (total inpatient service for a period x 100)/(total inpatient bed count x number of days in a period) [11].

Determination of daily defined dose (DDD)/100 bed days

DDD/100 bed days refer to the utilization of commonly used drug of different classes in the ICU for treatment of patients. This indicator is usually estimated when drug use by inpatients is taken into consideration [12].

DDD/100 bed days: (Number of units administered in a given period x 100)/(DDD x number of days x number of beds x occupancy index) [11].

Cost evaluation of antibiotic therapy

The cost of the antibiotic drugs was calculated from the cost list provided by the central pharmacy maintenance department of hospital, where the study was taking place. This data was used to estimate the cost of individual antibiotic therapy for each patient.

Statistical analysis

The data were collected and processed in MS Excel (Microsoft Corporation, Redmond, Washington). Statistical analysis was done using Microsoft Excel and GraphPad Prism, with results expressed as percentages and numbers wherever applicable.

## Results

Sociodemographic details of the study population

The study included 102 patients, including 57 males (54.88%) and 45 females (44.11%). The data provided shows the age-wise distribution of patients in the study population, with 34.31% of patients falling between the ages of 41 and 50, followed by those aged 51-60 years, making up 28.43% of the population (Table 1).

**Table 1:** Distribution based on sociodemographic profile of patients The data has been expressed as numbers and percentages.

Parameter	Number of Patients (N=102)	Percentage (%)
Gender		
Male	57	55.88
Female	45	44.11
Age distribution (in years)		
18-30	8	7.84
31-40	15	14.70
41-50	35	34.31
51-60	29	28.43
>61	15	14.70
Duration of hospitalization (in days)		
1-5	39	38.23
5-10	41	40.19
>10	22	21.56

Distribution based on disease condition

Based on the distribution done according to disease condition, it was determined that around 22 (21.56%) patients were affected by acute pulmonary oedema, followed by 20 (19.60%) patients with cerebral vascular attacks. Other common disease conditions were urinary tract infections, *Helicobacter pylori* infections, decompensated liver disease, tuberculosis, appendicitis, etc. (Table 2).

**Table 2:** Distribution based on presenting disease condition The data has been expressed as numbers and percentages.

Disease Condition	Number of Patients (N=102)	Percentage (%)
Acute pulmonary edema	22	21.56
Cerebrovascular accident	20	19.60
Pneumonia	9	8.82
Chronic kidney disease	6	5.88
Acute kidney injury	6	5.88
Decompensated liver disease	5	4.90
Sepsis	4	3.92
Appendicitis	3	2.94
Cerebral atrophy	3	2.94
Chronic obstructive pulmonary disorder	2	1.96
Hypoglycemia	2	1.96
Acute febrile illness	2	1.96
Cholelithiasis	2	1.96
Lower respiratory tract infection	2	1.96
Dengue	2	1.96
Tachycardia	2	1.96
*Helicobacter pylori* infection	2	1.96
Hepatic encephalopathy	1	0.98
Poisoning	1	0.98
Hernia	1	0.98
Drug reaction	1	0.98
Hypertension emergency	1	0.98
Tuberculosis	1	0.98
Lymphadenopathy	1	0.98
Urinary tract infection	1	0.98

Distribution based on antibiotic utilization

On determining the utilization of antibiotics, we observed that the most commonly prescribed antibiotic was a combination of injection Amoxicillin + Potassium clavulanate 101 (17.47%), followed by injection Sulbactam + Cefoperazone 94 (16.26%). Other prescribed antibiotics have been listed in Table 3. These antibiotics are commonly used to treat a variety of bacterial diseases, including respiratory, urinary tract, and skin infections. Cefotaxime 48 (8.30%), Meropenem 50 (8.65%), and Piperacillin + Tazobactam 57 (9.86%) were also commonly used, each with over 40 doses administered. Azithromycin was the least used antibiotic, with only 12 (2.07%) doses administered.

**Table 3:** Distribution of antibiotics utilization based on the dosage administration The data has been expressed as numbers and percentages.

Name of the Antibiotic	No of Dose	Percentage
Inj Amoxicillin + Potassium clavulanate	101	17.47
Inj Sulbactam + Cefoperazone	94	16.26
Inj Piperacillin + Tazobactam	57	9.86
Inj Vancomycin	54	9.34
Inj Clindamycin	54	9.34
Tab Rifaximin	54	9.34
Inj Meropenem	50	8.65
Inj Cefotaxime	48	8.30
Inj Ceftriaxone	40	6.92
Inj Tigecycline	14	2.42
Tab Azithromycin	12	2.07

During this analysis, we also determined that around 55% of antibiotics were prescribed empirically, 27% of antibiotics were given for prophylactic purposes, and around 18% were given definitively based on the culture sensitivity report. About 81.8% of antibiotics were given parenterally, and 18.2% were given orally. About 72% of patients, on average, were prescribed two antibiotics, and the rest were prescribed one antibiotic.

Estimation of daily defined dose (DDD)/100 bed days

The highest DDD per 100 bed days were for injection Tigecycline (J01AA12) at 15.5, followed by injection Sulbactam + Cefoperazone (J01RA17) at 10.4, which were significantly higher than the other antibiotics. This was indicative that, on average, patients receiving this antibiotic were being prescribed a higher dose or were prescribed for a longer duration than other antibiotics on the list (Table 4).

**Table 4:** Estimation of daily defined dose per 100 bed days for each antibiotic prescribed The data has been expressed as numbers and percentages. DDD: daily defined dose.

Name of the Antibiotic	ATC Code	No of Prescriptions	Percentage	DDD (in grams)	DDD/100 Bed Days
Inj Sulbactam + Cefoperazone	J01RA17	19	14	1	10.4
Inj Amoxicillin + Potassium clavulanate	J01CR02	17	12.7	2.2	5.1
Inj Piperacillin + Tazobactam	J01CR05	15	11	14	0.45
Inj Meropenem	J01DH02	15	11	3	1.85
Inj Ceftriaxone	J01DD04	13	9.7	2	2.2
Inj Vancomycin	J01XA01	12	9	2	3
Inj Cefotaxime	J01DD01	10	7.5	4	1.33
Inj Clindamycin	J01FF01	18	7.5	1.8	3.33
Inj Tigecycline	J01AA12	6	4.5	0.1	15.5
Tab Rifaximin	A07AA11	4	3	1.2	5
Tab Azithromycin	J01FA10	4	3	0.3	4.4

The occupancy index for the index was found to be 0.25 based on the information that the average inpatient service days per patient for 180 days was nine and that the total inpatient bed count was 20.

Estimation of cost for individual antibiotic therapy

The overall cost of the antibiotics used during the ICU stay for the given sample population varies greatly, ranging from ₹259.2/- to ₹59,596/-; the average cost incurred was ₹296.53/- per drug. The most expensive antibiotic was Magnex Forte, which was used for 90 ICU days, while the least expensive was Azithral, which was only used for 20 ICU days (Table 5).

**Table 5:** Estimation of cost for individual antibiotic therapy The data has been expressed as numbers. ICU: intensive care unit; IMCU: intensive medical care unit.

Name of the Antibiotic	Generic Name of Drug	Dose	Total Number of ICU Days	Cost Used for Antibiotics During IMCU Stay (Rs.)
Magnex Forte	Sulbactam + Cefoperazone	1.5 g	90	59,596
Tiganex	Tigecycline	50 mg	58	55,748
PIPTAZ	Piperacillin + Tazobactam	4.5 g	66	30,267
Dalacin C	Clindamycin	600 mg	104	18,252
Augmentin	Amoxicillin + Potassium clavulanate	1.2 g	72	13,130
Mero	Meropenem	500 g	75	11,296
Meromac	Meropenem	1 g	23	9,846
Anking	Vancomycin	500 mg	36	9,660
Anking	Vancomycin	1 g	42	3,696
Rifagut	Rifaximin	550 mg	35	2,278.8
Xone	Ceftriaxone	1 g	48	2,240
Taxim	Cefotaxime	1 g	66	1,632
PIPTAZ	Piperacillin + Tazobactam	2.25 g	1	350
Azithral	Azithromycin	500 mg	20	259.2
Total cost incurred				2,18,251

## Discussion

This six-month study evaluated antibiotics' use, sensitivity, and cost in a tertiary care teaching hospital's intensive medical care unit (IMCU). A total of 102 patient data points were collected prospectively and analyzed during the study period. The results indicated that 57 (55.88%) males and 45 (44.11%) females were admitted to the IMCU, which is identical to the study conducted by Marasine et al. [2].

According to the study's findings, 11 antibiotics, or an average of two antibiotics per patient, were provided during the study period. The development of an infection during a nosocomial stay, or surgery, and the presence of risk factors for infection brought on by numerous bacteria that are drug-resistant encourage increased antibiotic usage in the intensive care unit. In this study, 18% of antibiotic use was definitive, and 55% of antibiotic use was empirical [13]. Meropenem, Piperacillin + Tazobactum, and Sulbactam + Cefoperazone made up the majority of the empirically used antibiotics. This could be explained by the disease state of patients admitted to the intensive medical care unit (IMCU), where respiratory disorders were more prevalent in this study. For example, a delay in acquiring an antibiotic may be another reason to initiate empirical therapy to manage the status of admitted patients.

The majority of antibiotics prescribed were administered parenterally (82%), while oral administration was less common (18%). The IMCU treats a variety of sick patients who frequently find it difficult to take their medications orally. In this situation, parenteral preparations provide rapid onset action, higher absorption, and quick symptom alleviation while overcoming the challenges of oral administration. In addition to their advantages, they are also more likely to cause difficulties, make patients less comfortable, and cost more than oral formulations, raising the patient's overall healthcare costs. This finding was consistent with the results of the study done by Suraj et al. [11].

According to the results of the antibiotic use (DDD/100 bed days was 53), approximately half of the IMCU patients received one DDD of an antibiotic every day. This was significantly higher than a study carried out over a two-month period in a similar environment conducted by Marasine et al. [2]. The study on antibiotic utilization in the IMCU found the following patterns: Amoxicillin + Potassium clavulanate had a 12.7% prescription rate and a DDD of 5.1 g. Piperacillin + Tazobactam and Meropenem each accounted for 11% of prescriptions, with DDDs of 0.45 g and 1.85 g per 100 bed days, respectively. Sulbactam + Cefoperazone were prescribed in 14% of cases with a DDD of 10.4 g. Cefotaxime and Ceftriaxone were used in 7.5% and 9.7% of prescriptions, with DDDs of 1.33 g and 2.2 g, respectively. Vancomycin was used 9% of the time, with a DDD of 3 g. Rifaximin and Azithromycin were each prescribed four times, with DDDs of 5 g and 4.4 g. Tigecycline and clindamycin had 4.5% and 7.5% prescription rates, with DDDs of 15.5 g and 3.33 g, respectively. Overall, these antibiotics accounted for 52.56% of the total utilization, and these findings are consistent with the study done by Anand et al. [8].

The cost analysis of antibiotics used during IMCU stays revealed significant expenditures across various medications. Piperacillin + Tazobactam (PIPTAZ) incurred costs of ₹30,267 for 4.5 g used over 66 ICU days, and ₹350 for 2.25 g used once. Meropenem (MEROMAC) costs ₹9,846 for 1 g used over 23 ICU days and ₹11,296 for 500 mg used over 75 days. Sulbactam + Cefoperazone (Magnex Forte) totaled ₹59,596 for 1.5 g used over 90 ICU days. Cefotaxime (Taxim) costs ₹1,632 for 1 g used over 66 days, while Ceftriaxone (Xone) costs ₹2,240 for 1 g used over 48 days. Amoxicillin + Potassium clavulanate (Augmentin) cost ₹13,130 for 1.2 g used over 72 days. Vancomycin (Anking) amounted to ₹3,696 for 1 g used over 42 days and ₹9,660 for 500 mg used over 36 days. Tigecycline (Tiganex) was the most expensive at ₹55,748 for 50 mg used over 58 days, while Clindamycin (Dalacin C) cost ₹18,252 for 600 mg used over 104 days. Azithromycin (Azithral) cost ₹259.2 for 500 mg used over 20 days, and Rifaximin (Rifagut) totaled ₹2,278.8 for 550 mg used over 35 days. The total expenditure on antibiotics during IMCU stays amounted to ₹2,18,251, which on average accounted for ₹2,130.71 per patient, which is in range as suggested by the study done by Suraj et al. (₹1,403 per patient) [11] and Anand et al. (₹2,213 per patient) [8]. The pattern of antibiotic use in this study varies due to several factors, including a lack of appropriate drug use policies, processes and recommendations, and formulary books. Other factors that may contribute to antibiotic overuse and misuse in hospitals include inadequate monitoring, a lack of ongoing medical education, and a paucity of clinical pharmacologists or pharmacists. The limitations of this study was that it was conducted at a single site and a single center, with a limited number of patients; the outcome of the study was narrowed to understanding the antibiotic pattern and cost utilization at the specific location.

## Conclusions

Irrational or improper usage of antibiotics can lead to an increase in the financial burden of the patient. To minimize this burden, a well-planned antibiotic policy should be made and implemented to curb the irrational usage of antibiotics. In our study, we found that most of the antibiotics were prescribed empirically and that the majority of the prescriptions had two antibiotics. Amoxicillin + Potassium clavulanate were the most commonly prescribed antibiotics during the IMCU stay, and Magnex Forte, a combination of Sulbactam + Cefoperazone, was found to be the costliest drug. To have control over the increased economic burden of antibiotics, regular audits and inspections can be conducted. In the future, continuing medical education (CME) should be conducted to educate and train healthcare professionals to prescribe antibiotics rationally.
